# In systemic sclerosis TAPSE/sPAP ratio is correlated with ventilatory efficiency and exercise capacity assessed by CPET

**DOI:** 10.1007/s10238-022-00804-5

**Published:** 2022-02-12

**Authors:** Amalia Colalillo, Chiara Pellicano, Antonella Romaniello, Edoardo Rosato

**Affiliations:** 1 Department of Translational and Precision Medicine, Sapienza University of Rome, Viale dell’Università 37, 00185 Rome, Italy; 2 Division of Cardiology, Sant’Andrea Hospital, Rome, Italy

**Keywords:** Systemic sclerosis, Echocardiography, TAPSE/sPAP ratio, Cardiopulmonary exercise testing, Pulmonary hypertension

## Abstract

The aim of the study was to evaluate the correlation between cardiopulmonary exercise testing (CPET) parameters and right ventricular echocardiographic parameters for pulmonary arterial hypertension screening in a cohort of systemic sclerosis (SSc) patients. *Methods* forty SSc patients were examined using CPET and resting transthoracic echocardiography. CPET parameters analyzed were minute ventilation/carbon dioxide production (VE/VCO_2_) slope and maximum oxygen uptake (VO_2_ max); echocardiographic parameters were systolic pulmonary artery pressure (sPAP), tricuspid annular plane systolic excursion (TAPSE), and TAPSE/sPAP ratio. *Results* a positive correlation was observed between VE/VCO_2_ slope and age (*r* = 0.415, *p* < 0.01) and sPAP (*r* = 0.461, *p* < 0.01), conversely, a negative correlation was found between VE/VCO_2_ slope and TASPE/sPAP ratio (*r* = − 0.521, *p* = 0.001). VO_2_ max showed an inverse correlation with age (*r* = − 0.367, *p* < 0.05) and sPAP (*r* = − 0.387, *p* < 0.05) and a positive correlation with TAPSE/sPAP ratio (*r* = 0.521, *p* < 0.01). On stepwise linear regression analysis, VE/VCO_2_ slope was significantly correlated with TAPSE/sPAP ratio (*β* coefficient = − 0.570; *p* < 0.0001), as well as VO_2_ max was significantly correlated with TAPSE/sPAP ratio (*β* coefficient = 0.518; *p* = 0.001). *Conclusion* in SSc patients, TAPSE/sPAP ratio is the echocardiographic parameter of RV function which showed the best correlation with ventilatory efficiency and exercise capacity.

## Introduction

Pulmonary arterial hypertension (PAH) is characterized by progressive pulmonary vascular remodeling resulting in increased pulmonary vascular resistance and pulmonary arterial pressure, eventually leading to right ventricular (RV) failure [1]. PAH is a leading cause of morbidity and mortality in systemic sclerosis (SSc) patients [2]. Early diagnosis and treatment have been associated with improved outcomes [3, 4]. Annual screening with echocardiography, diffusing capacity of the lung for carbon monoxide (DL_CO_) and natriuretic peptides is recommended by current guidelines. Right heart catheterization (RHC) is required to confirm PAH diagnosis and to assess the severity of haemodynamic impairment [5]. Cardiopulmonary exercise testing (CPET) has been included in PAH diagnostic algorithm in order to reduce unnecessary invasive procedures [6, 7].

CPET is a safe non-invasive, dynamic technique providing an integrative assessment of cardiovascular, respiratory, metabolic, and muscular response to physical effort. Among CPET parameters, maximum oxygen uptake (VO_2_ max) and minute ventilation/carbon dioxide production (VE/VCO_2_) slope have been the most widely studied in cardiopulmonary diseases and have proved to be useful not only for PAH detection but also as prognostic markers [8, 9]. A low VO_2_ max is an index of reduced exercise capacity. A high VE/VCO_2_ slope reflects reduced ventilatory efficiency and is a hallmark of pulmonary vascular diseases [10].

Pressure overload is the main cause of RV dysfunction and failure in PAH. The RV–pulmonary arterial (PA) coupling describes the continuum of RV adaptation to pulmonary arterial load. The tricuspid annular plane systolic excursion/systolic pulmonary artery pressure (TAPSE/sPAP) ratio is the echocardiographic estimate of RV–PA coupling measured invasively by RHC [11]. A reduced TAPSE/sPAP ratio has been associated with poor outcomes in patients with PAH [12]. TAPSE/sPAP ratio is a powerful predictor of all-cause mortality in patients with moderate or severe tricuspid regurgitation [13]. Moreover, it has recently been demonstrated the potential role of TAPSE/sPAP ratio in SSc-PAH diagnosis [14].

An association between RV function parameters and VE/VCO_2_ slope and VO_2_ max has already been reported in heart failure and PAH [15, 16]. No study to date has specifically investigated the relationship between CPET measures and TAPSE/sPAP ratio.

The aim of the study was to evaluate the correlation between CPET parameters and RV echocardiographic parameters for PAH screening in a cohort of SSc patients.

## Methods

### Subjects

Forty patients meeting the 2013 American College of Rheumatology/European League against Rheumatism classification criteria for SSc [17] were enrolled in the study. Diffuse cutaneous SSc (dcSSc) and limited cutaneous SSc (lcSSc) were classified according to Le Roy et al. [18].

Patients with unstable angina, heart failure, arrhythmias, neurological disorders, valvular heart disease, uncontrolled arterial hypertension, hypertrophic cardiomyopathy, compromised exercise performance, mental or cognitive impairment, peripheral vascular diseases, anaemia, pregnant or breastfeeding women and subjects unable to give written informed consent were excluded. Calcium channel blockers, endotelin-1 receptor antagonist, and phosphodiesterase type 5 inhibitors were interrupted 72 h before CPET examination. CPET was performed 24 h prior to the next infusion of Iloprost.

The subjects’ written consent was obtained according to the declaration of Helsinki and the study was approved by the ethics committee of Sapienza University.

### Echocardiography

Resting transthoracic echocardiography was performed in all SSc patients by the same experienced operator with the General Electric Vivid S5 apparatus (GE Medical Systems, Israel Ltd.). Left ventricle (LV) diameter, wall thickness, LV ejection fraction (EF), RV diameter, TAPSE, left and right atrium area were assessed by standard methods [19]*.* sPAP was calculated from peak tricuspidal jet velocity using the simplified Bernoulli equation and combining this value with an estimate of the right atrium (RA) pressure: sPAP = 4(*V*)^2^ + RA pressure, where *V* is the peak velocity (in meters per second) of the tricuspid valve regurgitant jet, and RA pressure is estimated from inferior vena cava (IVC) diameter and respiratory changes [20].

### CPET

An incremental symptom-limited CPET was performed on an electronically braked cycloergometer (Ergoline-800, Mortara, Bologna, Italy), according to our previous study [21]. The subject was connected to the breath-by-breath lung gas exchange system by the use of a mask and breathing through a bidirectional turbine mass flow sensor (Quark PFT, Cosmed, Rome, Italy). The exercise protocol consisted of 3 min of rest and 3 min of unloaded cycling, followed by an incremental work rate to induce voluntary exhaustion in about 10 min, followed by 3 min of recovery. ECG and pulse oximetry were continuously monitored, and blood pressure was measured every two minutes. VO_2_, VCO_2_ and the respiratory exchange ratio (VCO_2_/VO_2_, RER) were computed and averaged every 10 s. The anaerobic threshold (AT) was determined by the *V*-slope method. The relation between VE and VCO_2_ (VE/VCO_2_ slope) was calculated as the slope of the linear relationship between VE and VCO_2_ from one minute after the beginning of the loaded exercise to the end of the isocapnic buffering period. A submaximal test is defined by RER ≤ 1.05.

### Statistical analysis

All results are expressed as the median and interquartile range (IQR), SPSS version 26.0 software was used for statistical analysis. The Shapiro–Wilk test was used to evaluate the normal distribution of data. Group of comparisons were made by Mann–Whitney test. The Fisher’s exact test was used to compare categorical variables. Spearman coefficient was used to evaluate the linear correlation. Stepwise regression analysis with significant variables in linear correlation was used to evaluate the correlation between the dependent variable (VE/VCO2 slope and VO2 max) and independent variables. *P*-values < 0.05 were considered significant.

## Results

Forty SSc patients [35 females (87.5%); median age 53 (IQR 46–59) years] were enrolled in the study. Twenty-three patients (57.5%) had lcSSc. Clinical, echocardiographic and CPET parameters of SSc patients are reported in Table 1.

**Table 1:** Clinical, echocardiographic and cardiopulmonary exercise testing (CPET) parameters of systemic sclerosis (SSc) patients

*Demographic and clinical features*	
Female, *n* (%)	35 (87.5)
Age, years	53 (46–59)
BMI, kg/m^2^	22.1 (20.4–24.5)
lcSSc, *n* (%)	23 (57.5)
Disease duration, years	11 (6–18)
SSc-specific autoantibodies, *n* (%)	
Scl70	17 (42.5)
Anti-centromere	19 (47.5)
None	4 (10)
Capillaroscopic pattern, *n* (%)	
Early	6 (15)
Active	13 (32.5)
Late	21 (52.5)
mRSS	11 (7–15)
DAI	1.5 (0.8–2.3)
DSS	4 (3–6)
FVC, % of predicted value	102 (92–108)
DLco, % of predicted value	73 (68–83)
*Echocardiographic parameters*	
Ejection fraction, %	60 (60–63)
Right atrium area, cm^2^	13.5 (9–19)
TRV, m/s	2.21 (1.53–3.68)
TAPSE, mm	24 (22–27)
sPAP, mmHg	28 (25–32)
TAPSE/sPAP mm/mmHg	0.87 (0.75–1.05)
*CPET parameters*	
RER	1.22 (1.14–1.31)
Maximum workload, W	80 (60–100)
VO_2_ max, mL/kg/min	19.7 (17.85–24.21)
VO_2_ max, % of predicted value	81 (73–87)
VO_2_@AT, mL/min	739 (651–887)
VE/VCO_2_ slope	29.2 (25.3–32.7)
PetCO_2_ max, mmHg	35 (33–38)
HR rest, bpm	79 (72–84)
HR max, bpm	155 (138–167)
SpO2 max, %	98 (96–99)

Values are *reported as median* (interquartile range). *BMI* Body mass index; *lcSSc* Limited cutaneous systemic sclerosis; *Scl70* Antitopoisomerase I antibodies; *mRSS* Modified Rodnan skin score; *DAI* Disease activity index; *DSS* Disease severity score. *FVC* Forced vital capacity; *DLco* Diffusing capacity of the lungs for carbon monoxide; *TRV* Tricuspid regurgitation velocity; *TAPSE* Tricuspid annular plane systolic excursion; *sPAP* Systolic pulmonary arterial pressure; *RER* Respiratory exchange ratio; *VO*_*2*_* max* Maximum oxygen uptake; *VO*_*2*_*@AT* VO_2_ at anaerobic threshold; *VE/VCO*_*2*_ Minute ventilation/carbon dioxide production; *PetCO*_*2*_ End-tidal carbon dioxide; *HR* Heart rate; *SpO*_*2*_ Arterial oxygen saturation

VE/VCO_2_ slope and VO_2_ max median values were 29.2 (IQR 25.3–32.7) and 19.7 (IQR 17.85–24.21) mL/min/kg, respectively. sPAP and TAPSE/sPAP ratio median value were 28 (25–32) mmHg and 0.87 (IQR 0.75–1.05) mm/mmHg, respectively.

**Figure 1:**
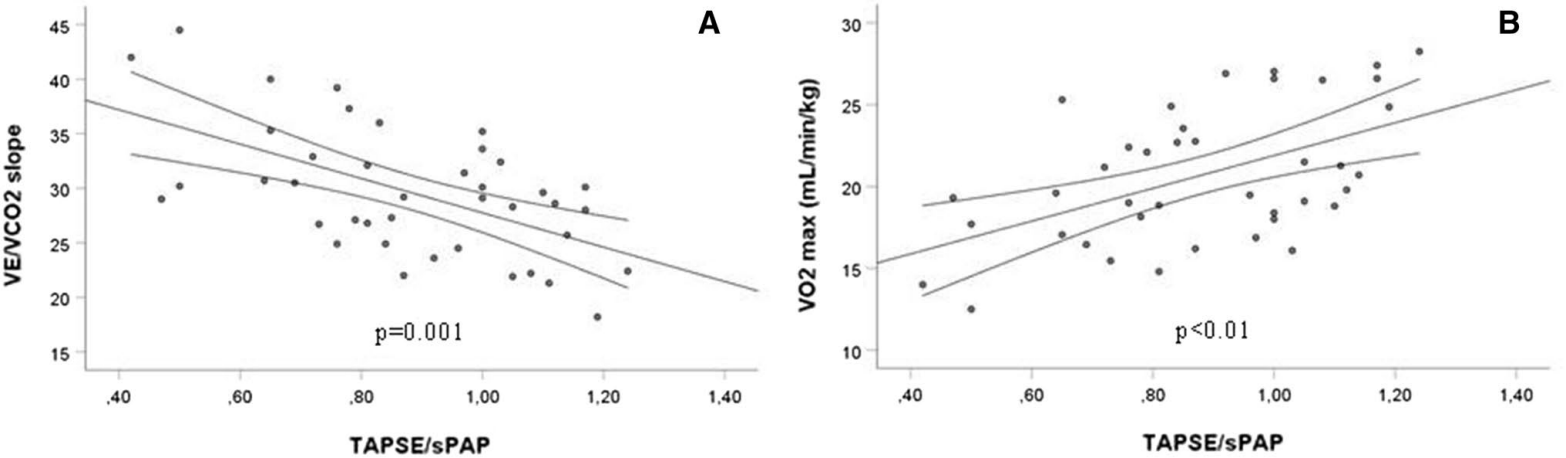
**A** Correlation between minute ventilation/carbon dioxide production (VE/VCO_2_) slope and tricuspid annular plane systolic excursion/systolic pulmonary artery pressure (TAPSE/sPAP) ratio; **B** Correlation between maximum oxygen uptake (VO_2_ max) and TAPSE/sPAP ratio

A significant positive correlation was found between VE/VCO_2_ slope and age (*r* = 0.415, *p* < 0.01) and sPAP (*r* = 0.461, *p* < 0.01), conversely, a significant negative correlation was observed between VE/VCO_2_ slope and TAPSE/sPAP ratio (*r* = − 0.521, *p* = 0.001) (Fig. 1). No correlation was found between VE/VCO_2_ slope and TAPSE. By stepwise linear regression analysis, VE/VCO_2_ slope was significantly correlated with TAPSE/sPAP ratio (*β* coefficient = − 0.570; *p* < 0.0001).

VO_2_ max showed a significant negative correlation with age (*r* = − 0.367, *p* < 0.05) and sPAP (*r* = − 0.387, *p* < 0.05) and a significant positive correlation with TAPSE/sPAP ratio (*r* = 0.521, *p* < 0.01) (Fig. 1). No correlation was found between VO_2_ max and TAPSE. On stepwise regression analysis VO_2_ max was significantly correlated with TAPSE/sPAP ratio (*β* coefficient = 0.518; *p* = 0.001).

## Discussion

In this study, VE/VCO_2_ slope and VO_2_ max showed a correlation with age, sPAP and TAPSE/sPAP ratio. Therefore, in multiple regression analysis TAPSE/sPAP ratio is the only variable, which shows a significant correlation with exercise capacity (VO_2_ max) and ventilatory efficiency (VE/VCO_2_ slope).

Reducing the time to PAH diagnosis can significantly improve patients’ survival. Current guidelines recommend resting echocardiography as a screening test in SSc patients. Although elevated sPAP values are associated with an increased probability of PAH, sPAP estimation may be inaccurate in the individual patient. In the ESC/ERS guidelines, CPET was performed to reduce unnecessary invasive procedures and in PAH risk assessment [5].

In our study, we demonstrated that TAPSE/sPAP ratio is the best echocardiographic parameter of RV function which correlates with VE/VCO_2_ slope and VO_2_ max. In clinical practice, sPAP is the most used echocardiograpic parameter to evaluate PAH. In our previous study, we demonstrated that in SSc patients with a DETECT algorithm step 2 total score > 35 TAPSE/sPAP ratio can be used to further select patients requiring RHC to confirm PAH diagnosis [14]. Dumitrescu et al*.* investigated the role of CPET in early PAH detection in a prospective study of 173 SSc patients, without known PAH, who also underwent RHC. PeakVO_2_ and VE/VCO_2_ were the CPET parameters, which showed the highest correlations with pulmonary haemodynamics. PeakVO_2_ was the most accurate parameter for diagnosis (sensitivity 87.5%, specificity 74.8% at a threshold level of 13.8 mL/min/kg) and a peakVO_2_ of > 18.7 mL/kg/min excluded PAH (negative predictive value of 1.0). In addition, a nadir VE/VCO_2_ ratio of > 45.5 showed a positive predictive value of 1.0 [6]. Santaniello et al. demonstrated that CPET is useful, in addition to the DETECT algorithm, to select patients for RHC referral. Among CPET parameters VE/VCO_2_ slope showed the best performance to predict PAH at RHC [7]. An association between RV function parameters and VE/VCO_2_ slope and VO_2_ max has already been reported. In heart failure, higher sPAP and lower RV fractional area change (FAC) were correlated with worse percent predicted VO_2_ max and higher VE/VCO_2_ slope [15]. In PAH, resting RV function assessed by FAC or RV longitudinal strain was also associated with the CPET parameters of exercise capacity and ventilatory efficiency [16]. To date, no study has investigated the correlation between TAPSE/sPAP ratio and CPET parameters. We can assume that TAPSE/sPAP ratio could be a useful echocardiographic parameter of RV function to select SSc patients requiring CPET. The main limitations of our study are the small sample size, the lack of a control group, the lack of RHC data and the prospective role of TAPSE/sPAP ratio during the follow-up of SSc patients.

In conclusion, in SSc patients TAPSE/sPAP ratio is the echocardiographic parameter of RV function which showed the best correlation with ventilatory efficiency and exercise capacity. Future larger studies are needed to confirm our preliminary data.
